# Variability in muscle function deficits and clinical identification of sarcopenic obesity in older adults with type 2 diabetes

**DOI:** 10.3389/fmed.2025.1747916

**Published:** 2026-02-18

**Authors:** Sharon Barak, Roy Eldor, Idit Dotan, Roy Brown, Elena Izkhakov, Yona Greenman, Carla M. Prado, Assaf Buch

**Affiliations:** 1 Department of Nursing, School of Health Sciences, Ariel University, Ariel, Israel; 2 Department of Pediatric Rehabilitation, The Edmond and Lily Safra Children's Hospital, The Chaim Sheba Medical Center, Ramat-Gan, Israel; 3 Institute of Endocrinology, Metabolism and Hypertension, Tel Aviv Sourasky Medical Center, Tel Aviv, Israel; 4 Sackler Faculty of Medicine, Tel Aviv University, Tel Aviv, Israel; 5 Institute of Endocrinology, Diabetes and Metabolism, Rabin Medical Center, Beilinson Hospital, Petah Tikva, Israel; 6 Geriatric Division, Tel Aviv Sourasky Medical Center, Tel-Aviv, Israel; 7 Department of Agricultural, Food and Nutritional Science, Human Nutrition Research Unit, University of Alberta, Edmonton, AB, Canada; 8 Department of Nutritional Sciences, School of Health Sciences, Ariel University, Ariel, Israel

**Keywords:** diagnostic criteria, muscle function, older adults, sarcopenic obesity, type 2 diabetes

## Abstract

**Introduction:**

Sarcopenic obesity (SO) is increasingly recognized as a major yet often overlooked complication in older adults—particularly those with type 2 diabetes (T2D)—and is linked to adverse events. SO evaluation requires muscle function tests [handgrip strength (HGS), knee extensor strength, chair-stand] and body composition. However, SO prevalence can vary widely depending on the muscle function test used, and these assessments are not routinely implemented in clinical care as they might be time-consuming.

**Aims:**

(1) Evaluate differences in the prevalence of altered muscle function using internationally accepted clinical guidelines among community-dwelling older adults with T2D; (2) to develop risk clusters for predicting increased SO probability; and (3) to develop an SO index based on routine clinical measures that differentiates individuals by their likelihood of having SO.

**Methods:**

Participants underwent comprehensive assessments of muscle function, including HGS, maximal knee extension strength, and chair stand test. Body composition was evaluated using bioelectrical impedance analysis to determine appendicular lean mass relative to body weight. Clinical data, including waist circumference, body mass index, prescription medications, metabolic markers, sex hormones, physical function tests, dietary intake, and physical activity, were collected using standardized protocols. Prevalence of altered muscle function and SO obesity was calculated using internationally accepted SO criteria.

**Results:**

Participated in the study 100 community dwelling older adults diagnosed with T2D (mean age: 69.75 ± 4.64). Altered muscle function prevalence ranged from 1% (HGS) to 92% (chair-stand). SO was present in 72% of the sample. Cluster analysis identified three SO severity groups: severe (*N* = 37), moderate (*N* = 32), and mild (*N* = 31). Significant differences in health, sex hormones, and physical function were noted across clusters. A SO risk index using five routine clinical measures (waist circumference, body mass index, medication count, HbA1c, and chair stand) effectively distinguished SO risk clusters (AUC = 0.87).

**Conclusion:**

Among older adults with T2D, SO was prevalent, with substantial impairments in muscle function and body composition. Disease severity was primarily driven by alterations in appendicular lean mass and lower-limb strength, whereas handgrip strength showed limited discriminatory capacity. These findings highlight the importance of comprehensive functional and body composition assessment for older adults with T2D.

**Clinical trial registration:**

clinicaltrials.gov, identifier: NCT03560375.

## 1 Introduction

Diabetes-related sarcopenia is a significant contributor to the global prevalence of sarcopenia (1). Accordingly, in a meta-analysis of studies evaluating the prevalence of sarcopenia in diabetes in the community-dwelling general population aged ≥ 60 years, the prevalence of sarcopenia was significantly higher in diabetics (15.9%) than non-diabetics (10.8%) (2). Similarly, a more recent study reported that sarcopenia was present in 14.1% of both elderly and non-elderly adults with type 2 diabetes (T2D) (1). T2D is also associated with an increased risk of developing sarcopenic obesity (SO). In addition to T2D, numerous other factors are associated with SO. For example, SO is associated with insulin resistance, inappropriate nutrition, increased body fat, and physical functioning difficulties. In contrast, high physical fitness and activity levels are related to a decreased SO risk (3).

SO is associated with adverse health outcomes, including increased risks of falls, disability, hospitalization, metabolic and cardiovascular morbidity, and premature mortality (4, 5). Importantly, SO represents a “double burden” syndrome, as both obesity and sarcopenia independently worsen health status, but their combination exerts a synergistic effect on frailty and health care utilization (6). Given the adverse implications of SO and the high prevalence of SO among individuals with T2D, early detection of SO susceptible groups and creation of SO risk clusters based on the known SO risk factors is essential for selecting appropriate interventions to reduce the occurrence of it and various adverse outcomes in this demographic (7).

To promote SO research and clinical care, the European Society for Clinical Nutrition and Metabolism (ESPEN) and the European Association for the Study of Obesity (EASO) have published SO diagnostic criteria. According to the proposed criteria, diagnostic procedures should include: (1) assessment of skeletal muscle function – based on various tests, including maximal handgrip strength (HGS), knee extensor strength, or the chair-stand test; and (2) assessment of body composition to measure fat and lean/muscle mass (e.g., skeletal muscle mass; body mass index, BMI; fat mass) (4).

The assortment of proposed techniques for assessing skeletal muscle alterations and body composition could potentially impact the prevalence of SO (7, 8).

Variability in skeletal muscle alterations reflects differences across muscle function tests, which assess distinct domains of strength and endurance. Specifically, HGS evaluates upper-limb isometric strength, knee extensor tests assess lower-limb strength, and the chair-stand test reflects lower-limb muscular endurance, with weak correlations observed between upper-limb strength and lower-limb endurance (9). Second, the systemic nature of T2D related musculoskeletal impairments suggests that lower and upper extremity changes occur concurrently. However, the association between the magnitude of changes in function and muscle strength is only moderate (10). Another possible caveat with the proposed comprehensive SO diagnostic procedure is its requirement for specialized equipment (e.g., handgrip dynamometer), which is not easily accessible in all clinics. In addition, assessment requires time. Therefore, during patient visits in primary care, providers rarely perform a comprehensive screen for indicators, such as slowness of gait, due to growing administrative burden limiting patient contact time. Therefore, there is a need to develop a SO risk index score utilizing simple commonly used measures in clinical settings.

The aims of the current study were to: (1) evaluate differences in prevalence of altered muscle function using the ESPEN/EASO recommended tests (HGS, knee extensor strength, the chair-stand test) among community-dwelling older adults with T2D; (2) to develop risk clusters for predicting increased probability for SO in this population; and (3) to design a SO index that utilizes routinely used clinical measures to distinguish between individuals with different probabilities to be diagnosed with SO. It was hypothesized that using different ESPEN/EASO tests, altered muscle function prevalence will vary, with HGS showing the lowest altered muscle function rate. Moreover, distinct severity clusters of SO can be identified based on muscle function and body composition measures, with significant differences in demographic and clinical characteristics across these clusters (e.g., older participants in the more severe groups). To test the study’s hypotheses a cross-sectional study was employed. Enhancing knowledge on SO evaluation and prediction is crucial, as varying screening tests may lead to different SO diagnoses status, thereby influencing the prevention and treatment programs offered to these individuals (10).

## 2 Materials and methods

### 2.1 Subjects

**Figure 1:**
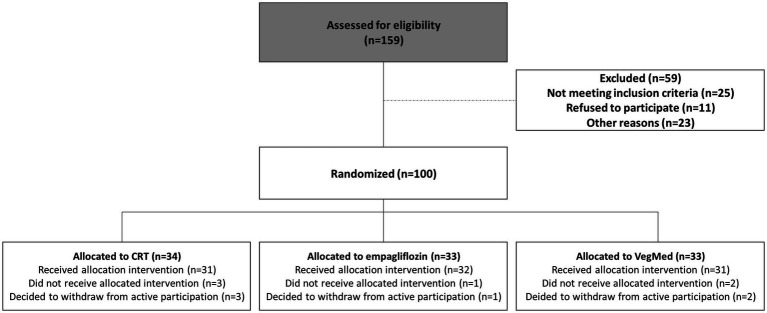
Study participants’ CONSORT flow diagram. This study is composed of a secondary analysis of a trial examined lifestyle and pharmacotherapy effects in older adults with type 2 diabetes. Eligible participants had type 2 diabetes, obesity, and low physical activity, excluding those with severe comorbidities. For the purpose of the study baseline data from the three intervention groups were used: CRT, circuit resistance training; VegMeD, vegetarian/Mediterranean diet.

This study was based on a secondary analysis of a randomized clinical trial, the CEV-65 trial (11). The trial aimed to assess the efficacy of 10 weeks effects of a modified plant-based Mediterranean (vegeterranean) diet, circuit resistance training, and empagliflozin, alone or in combination, on metabolic and physical function in older adults with T2D. Main eligibility criteria for CEV-65 were confirmed T2D, obesity (waist circumference [WC] or BMI), treated with non-SGLT-2 antihyperglycemics, HbA1C 6.5–8%, and low physical activity level (perform <2 days a week of any leisure aerobic physical activity). Exclusions included severe kidney disease (estimated glomerular filtration rate <45 mL/min), severe cardiovascular issues, advanced neuropathy, resistance training, recent diet changes, or weight loss. Additionally, individuals treated with SGLT-2 inhibitors were excluded at recruitment to avoid pharmacological overlap and contamination with the empagliflozin intervention arm. Although the current analysis focuses solely on baseline characteristics, maintaining the original inclusion and exclusion criteria was necessary to preserve cohort homogeneity and methodological consistency with the parent study design. The CONSORT flow diagram illustrating participant recruitment, randomization, and allocation to the original study intervention arms is presented in Figure 1. Subjects were recruited from the hospital’s Endocrinology, Metabolism, Hypertension and Diabetes unit and from primary care physician clinics in the center of the country.

### 2.2 Outcome measures

#### 2.2.1 Alterations in body composition (muscle mass)

Alterations in body composition were established according to the ESPEN/EASO suggestion to adopt the cut points used by Batsis et al., (12) for appendicular lean mass adjusted to body weight (ALM/W). According to the reference cut points provided for males and females ≥ 60y mixed ethnicity, the value of 2 standard deviations below the mean of a healthy young reference group was used (< 25.7% for males and < 19.4% for females). Assessments were conducted using bioelectrical impedance analysis technology (BIA; *InBody 770 body composition analyzer, InBody Co., Ltd, Seoul, Korea*) technology using propriety equation.

#### 2.2.2 Alterations in muscle function (sarcopenia)

HGS was measured using a hand-held dynamometer (*Jamar® Sammons Preston Rolyan, Chicago, Illinois, USA*). Subjects were seated upright with their arms at their sides and elbows bent at 90°. Demonstrations were conducted for each hand, followed by three alternating trials between arms. The highest value from the six measurements was recorded (13). Impaired HGS was defined as maximal strength < 27 kg and < 16 Kg for males and females, respectively (4).

Maximal knee strength was measured using an isometric knee extension test with a hydraulic Push-Pull Dynamometer (*Baseline® Evaluation Industries*). Three 5-s maximum effort measurements were taken per leg, with 60-s pauses between trials. The highest of the six measurements was recorded (14). Impaired knee extension strength defined as maximal knee extension strength/total weight < 0.4 and <0.31 kg/kg for males and females, respectively (4).

Lower extremity muscular endurance was measured using the STS30. Participants practiced one or two repetitions to ensure proper form and balance. Upon the signal “go,” participants were instructed to rise to a full standing position and then return to a seated position as many times as possible within 30 s. The test consisted of one trial (15). Impaired STS30 was categorized based on age and sex starting from <17–15 repetitions up to <13–12 repetitions (4).

#### 2.2.3 Clinical characteristics

Health status and clinical data were collected through subjective questions regarding personal health perception, adapted from the MABAT survey (16). Detailed information on prescription medication use was gathered by the research team using a structured questionnaire based on the MABAT survey and further verified through additional questioning by the research physician during the medical anamnesis. Additionally, WC and weight and height measurements (BMI) were taken according to a uniform protocol. Further clinical measurements included endocrine blood tests, which assessed sex hormones: testosterone (total and bioavailable in serum using the Electrochemiluminescence method), FSH (in serum using the Immunochemiluminescence method), and estradiol (in plasma using the Electrochemiluminescence method). IGF-1 (measured in serum using the Immunochemiluminescence method) and vitamin D (measured in serum using the ELISA method) levels were also evaluated. Vitamin D status was defined as deficient (< 12 ng/mL; < 30 nmol/L), insufficient (12–30 ng/mL; 30–75 nmol/L), or sufficient (≥ 30 ng/mL; ≥ 75 nmol/L), according to commonly used clinical cut-off values (17). All these tests, including the insulin test (detailed below), were performed in the Endocrine Laboratory at the hospital according to uniform protocols.

Diabetic specific measures were assessed through a medical interview and according to the medical record. Fasting blood tests were conducted according to standard protocols and included: blood glucose, HbA1c, measured on a G8 TOSOH HPLC; fasting morning insulin, measured in plasma using the Electrochemiluminescence COBAS 601 (*Roche Diagnostics*).

Physical function assessment was conducted using a variety of well-established tests for evaluating functionality in older adults (4). The performed tests included the Timed Up and Go test (TUG), and the 2-min walk test. For subjective functional assessment, the Comprehensive Functional Assessment Questionnaire (18) was used. This questionnaire evaluates physical function through questions on activities of daily living. A higher score, ranging from 0 to 36, indicates lower functional ability, with a score of 16 of higher signifying objective functional impairment (19).

Metabolism and healthy lifestyle included the measurement of resting metabolic rate and respiratory quotient as described previously (20), and the evaluation of dietary intake (kcal/day, protein intake and total nutrition score) using a 24-h recall questionnaire. The data were analyzed using T*he Israel Ministry of Health’s “Tzameret” dietary analysis program* (21). Smoking status was also evaluated via participants’ self-report. Finally, physical activity levels were assessed based on a detailed interview using the MABAT survey questionnaire (16). The questionnaire captured frequency, duration, intensity, and long-term engagement across a wide range of physical activity types (e.g., walking, running, cycling, swimming, aerobic exercise, resistance training, and others). Specifically, participants reported the frequency of each activity (times per week), duration (minutes per session), and intensity (light, moderate, or vigorous effort, defined by perceived exertion such as accelerated breathing and sweating). In addition, participants reported the length of continuous engagement in each activity (from <6 months to several years). Total energy expenditure was calculated using the American College of Sports Medicine metabolic equations and presented as Kcal/week (22).

### 2.3 Statistical analyses

#### 2.3.1 Power analysis

Sample size was calculated using G*Power Statistical Power Analyses for Windows (Version 3.1.9.6, Heinrich Heine Universität Düsseldorf). A calculation was made for comparison between three independent groups with a two-sided alpha level of 0.05, a 1:1:1 group ratio, a medium effect size (Cohen’s d = 0.5), and a power of 80%. These data determined a sample size of 74 participants. For the purpose of this study, only baseline data were used from all three-study groups in a phase where there was allocation concealment.

#### 2.3.2 Prevalence of altered muscle function, body composition, and SO

Prevalence of participants presenting/not presenting with altered muscle function when using each of the recommended muscle alteration tests (HGS, knee strength, and STS30) was calculated and compared using chi-squared tests. The prevalence of muscle alterations based on one or more functional tests, as well as the prevalence of abnormal body composition and SO, were also calculated.

#### 2.3.3 Sarcopenic obesity severity clusters

SO severity clusters were identified based on muscle function and body composition, using mean handgrip strength values because only one participant showed impairment on this test. Next, a two-step cluster analysis was conducted (23). In the initial step, a sequential approach was employed to pre-cluster the cases by defining dense regions within the attribute space under analysis. The procedure is implemented by constructing a modified cluster feature (CF) tree. If the CF tree grows beyond allowed maximum size, the CF tree is rebuilt based on the existing CF tree by increasing the threshold distance criterion. This process continues until a complete data pass is finished (24). An outlier-handling step was incorporated during construction of the CF tree. Outliers were defined as data records that did not fit well into any cluster and were identified at the leaf level when the number of records in an entry was less than 25% of the size of the largest leaf entry. Potential outliers were temporarily removed prior to rebuilding the CF tree and subsequently reassessed to determine whether they could be reintegrated without increasing tree size. Entries that remained small and could not be accommodated at the end of CF tree construction were classified as outliers. Subsequently, these pre-clusters underwent statistical merging in a stepwise manner until all clusters converged into one (25). The process starts by defining a starting cluster for each of the sub-clusters produced in the pre-cluster step. All clusters are then compared, and the pair of clusters with the smallest distance between them is selected and merged into a single cluster. After merging, the new set of clusters is compared, the closest pair is merged, and the process repeats until all clusters have been merged. A cut-off of 80% was set to determine unfavorable results (26) indicating that over 80% of the sample exhibited unfavorable alterations in body composition and in each muscle function test utilized (excluding HGS). Based on the number of unfavorable risk factors within each cluster, clusters were categorized as follows: “severe condition” (comprising 3–4 unfavorable outcomes), “moderate condition” (with 2 unfavorable outcomes), and “mild condition” (0–1 unfavorable conditions).

Finally, characteristics of the clusters were evaluated and compared. Continuous variables were evaluated using a one-way analysis of variance with the Tukey–Kramer post-hoc test. Categorical variables were evaluated using the chi-squared test. Differences in sex hormone levels across SO obesity severity clusters were examined using analysis of covariance (ANCOVA). Separate models were fitted for females and males. SO cluster membership was entered as a fixed factor, while age, BMI, HbA1c were included as covariates to control for potential confounding. Assumptions of ANCOVA, including homogeneity of variances, homogeneity of regression slopes, and normality of residuals, were evaluated and met. Adjusted marginal means and standard errors were estimated for each cluster, and overall between-cluster effects were assessed using *F* statistics. When significant main effects were observed, Bonferroni-adjusted *post hoc* comparisons were conducted. Statistical significance was set at *p* < 0.05.

#### 2.3.4 Sarcopenic obesity risk index

**Figure 2:**
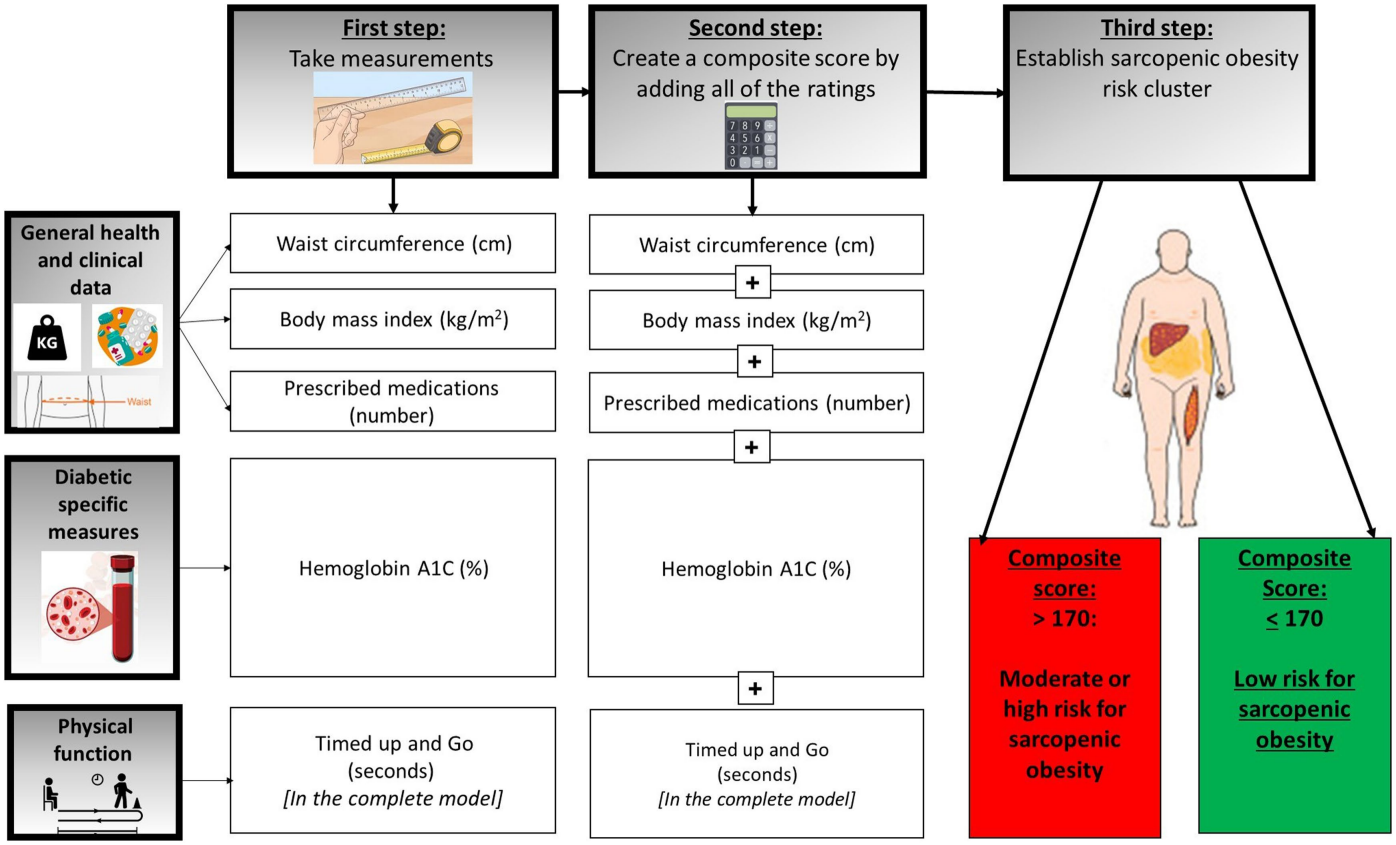
Calculation of sarcopenic obesity risk index. Construction of sarcopenic obisity risk groups – risk groups classification is based on the presence of alterations in body composition (specifically appendicular mass) and muscle function (maximal knee strength test, lower extremity muscular endurance test, and mean maximal handgrip strength). A cut-off of 80% was set to determine unfavorable results, indicating that over 80% of the sample exhibited unfavorable alterations in body composition and in each muscle function test utilized (excluding handgrip test). Based on the number of unfavorable risk factors, sarcopenic obesity risk groups were categorized as follows: “severe condition” (comprising 3–4 unfavorable outcomes), “moderate condition” (with 2 unfavorable outcomes), and “mild condition” (0–1 unfavorable conditions).

To formulate an SO risk index, variables showing statistically significant differences between clusters were aggregated, excluding: (1) the two-minutes’ walk test and physical activity level, both of which moderately correlated with TUG (with correlation coefficients ranging between 0.50–0.70); and (2) variables not commonly available in routine clinical practice (FSH, estradiol, insulin, insulin resistance) were omitted. Two SOB risk indexes were constructed. The first, the complete SOB risk index, comprised five variables: WC, BMI, number of prescribed medications, HbA1c, and TUG, where a higher score indicates a greater risk of SOB. However, as during patient visits in primary care, providers rarely perform a comprehensive screen for indicators, such as slowness of gait, a reduced score was also calculated comprising only four indicators, namely, WC, BMI, HbA1c, and number of prescribed medications. Regarding the later, medication counts were restricted to predefined drug families directly relevant to cardiometabolic health and T2D management. These included lipid-lowering medications (statins, ezetimibe, and PCSK9 inhibitors), antiplatelet agents used for thrombosis prevention, and cardiovascular and antihypertensive therapies (beta-blockers, calcium-channel blockers, and angiotensin-converting enzyme inhibitors or angiotensin receptor blockers). In addition, glucose-lowering treatments were recorded and categorized as oral agents (metformin, thiazolidinediones, dipeptidyl peptidase-4 inhibitors, and sulfonylureas or glinides) and injectable therapies (glucagon-like peptide-1 receptor agonists and insulin therapy, including long- and short-acting formulations). Only medications within these clinically relevant categories were included, while supplements and therapies unrelated to cardiometabolic or diabetes care were excluded. Medications were counted individually, even when multiple drugs from the same therapeutic class were prescribed, to reflect overall pharmacological burden, which serves as a clinically meaningful proxy for disease severity and treatment complexity in older adults (27). For illustration of the SOB risk index calculation, refer to Figure 2.

The accuracy of the SO risk index to discriminate between individuals with high and moderate risk vs. low risk was evaluated using receiver operating characteristic (ROC) analysis (28). The ROC area under the curve (AUC) was interpreted as follows: 0.5 < AUC < 0.7 is less accurate, 0.7 < AUC < 0.9 is moderately accurate, 0.9 < AUC < 1.0 is very accurate, and AUC = 1.0 is perfectly accurate (29). Criterion score, sensitivity and specificity were also calculated.

In all analyses, except for ROC analysis, the level of significance was set to *p* < 0.05 (two-tailed) using IBM SPSS Statistics (version 29.0). ROC analysis was conducted using MedCalc 14.8.1 Statistical Program (MedCalc Software, Ostend, Belgium).

## 3 Results

One hundred community dwelling older adults diagnosed with T2D participated in this study (mean age: 69.75, interquartile range: 67.00–73.00; 60% females). Additional sociodemographic and clinical characteristics of the study participants are presented in Table 1.

**Table 1:** Study participants’ characteristics.

Assessment domain		Variables		Total group (*N* = 100): Mean ± SD or median [IQR] or *N* (%)
Socio-demographic characteristics		Age		69.75 [67.00–73.00]
		Sex	Females	60 (60.0)
			Males	40 (40.0)
		School years		15.00 [13.00–16.00]
		Economic status	Not that good/moderate	30 (30.0)
			Good/excellent	70 (70.0)
		Marital status	Living alone	39 (39.0)
			A relationship	61 (61.0)
Clinical characteristics	General health	Health perception	Not that good/not good at all	42 (42.0)
			Good and very good	58 (58.0)
	Diabetic specific measures	Age diabetes		56.94 ± 9.30
		Glucose (mg/dL) (*n* = 85)		137.50 [119.45–162.20]
		HbA1c (%) (*n* = 97)		7.30 [6.60–7.90]
		Insulin resistance, HOMA-IR (*n* = 78)		4.64 [2.61–7.55]
Anthropometrics and body composition		Waist circumference (cm)		107.25 [100.00–117.50]
		Waist circumference – exceeding healthy thresholds		93 (93.0)
		Body mass index (kg/m^2^)		31.7 ± 5.96
		Body mass index (kg/m^2^) – exceeding healthy thresholds		59 (59.0)
		Fat percentage		40.75 ± 7.25
		Fat percentage – exceeding healthy thresholds		62 (62.0)
		ASLT/weight (kg/kg)		24.08 ± 3.38
		ASLT/weight – below healthy thresholds		73 (73.0)
Physical function		Timed Up and Go (seconds)		10.55 [9.45–11.82]
		2 Minutes’ walk test (meters)		162.83 ± 27.25
		Sit to Stand (times)		11.00 [10.00–12.00]
		Maximal hand grip (kg)		28.95 [23.90–37.60]
		Maximal knee extensor strength (kg/kg)		29.14 ± 9.69

ASLT, Appendicular soft lean tissue; HOMA-IR, Homeostatic Model Assessment for Insulin Resistance; IQR, interquartile range; SD, standard deviation.

### 3.1 Prevalence of altered muscle function, body composition, and sarcopenic obesity

The prevalence of altered muscle function varied across the three tests employed, with 1, 49, and 92% of the sample showing altered muscle function in HGS, maximal knee strength, and STS30, respectively. Remarkably, 51, 44, and 1% of the sample displayed altered muscle function in just one, two, and all three tests utilized to assess altered muscle function, respectively. Merely 4% of the sample showed no muscle function alteration in any of the tests conducted (Supplementary Figure 1). Moreover, overall 72% exhibited concurrent alterations in body composition and muscle function consistent with SO.

Cluster analysis reported a three-cluster classification solution (Silhouette = 0.6) with smallest (*N* = 31, Cluster 3) to largest (*N* = 37, Cluster 1) ratio of 1.19. Following the risk stratification labeling outlined in the statistical analysis section, clusters were delineated based on SO severity. In the severe cluster (cluster 1, *N* = 37), all participants exhibited alterations in body composition, maximal knee strength, and STS30. Additionally, the mean HGS in this cluster was comparable to that observed in the moderate and mild severity groups (*F* = 1.79, *p* = 0.17). Within the moderate severity cluster (cluster 2, *N* = 32), all participants displayed alterations in body composition and abnormal STS30. However, all participants maintained normal maximal knee strength, with similar HGS to other severity groups. Finally, in the mild severity group, 16.10% (alterations in body composition) to 38.79% (altered maximal knee strength) presented alterations in body composition or muscle function, while HGS remained similar to the other severity groups. Prevalence of altered muscle function and body composition in each of the three clusters are summarized in Table 2.

**Table 2:** Sarcopenic obesity severity clusters among older adults with type 2 diabetes.

Evaluation domain	Assessment	Cluster 1 (*N* = 37): Severe condition	Cluster 2 (*N* = 32): Moderate condition	Cluster 3 (*N* = 31): Mild condition	Chi-squared test (*p* value) or *F*-test (*p* value)
Alterations in body compositions	Appendicular mass^1^: %	100.00^c^	100.00^c^	16.10^a,b^	49.62 (<0.001)
Alterations in muscle function	Maximal knee strength^2^: (%)	100.00^b,c^	0.00^a,c^	38.70^a,b^	68.00 (<0.001)
	Lower extremity muscular endurance^3^: (%)	100.00	100.00	25.80	36.80 (<0.001)
	Maximal hand grip: mean (SD)	29.22 (8.01)	30.74 (9.76)	33.41 (9.76)	1.79 (0.17)

^a^Statistically significantly different from “Cluster 1” (2-tailed; *p* < 0.05); ^b^Statistically significantly different from “Cluster 2” (2-tailed; *p* < 0.05); ^c^Statistically significantly different from “Cluster 3” (2-tailed; *p* < 0.05); SD, standard deviation.

^1^Appendicular mass (lean mass in upper and lower limbs) / total weight < 25.7% for males and < 19.4% for Females (12).

^2^Maximal knee strength - Impaired knee extension strength defined as maximal knee extension strength/ total weight < 0.4 and <0.31 kg/kg for males and females, respectively (4).

^3^Lower extremity muscular endurance – Impaired function was categorized based on age and sex starting from <17–15 repetitions up to <13–12 repetitions.

^4^Maximal handgrip – Impaired handgrip was defined as maximal strength < 27 kg and < 16 Kg for males and females, respectively (4). The average value was used as most participants presented normal handgrip.

Concerning the assessment of cluster predictors’ significance, alterations in appendicular mass and maximal knee strength stood out as the most prominent predictors, with respective importance scores of 1 and 0.9. Following was alterations in STS30 (predictor importance score = 0.3). Conversely, HGS was deemed the least influential predictor (predictor importance score of = 0.1).

### 3.2 Between-clusters differences in demographic and clinical characteristics

Between-cluster differences in demographic, anthropometric, metabolic, medication, physical function, and physical activity variables are presented in Table 3. No differences between clusters were noted in any of the demographic and metabolic characteristics (range of *F*: 0.02 to 1.65, range of *P*: 0.19 to 0.97). However, significant differences between clusters were observed in various general and diabetic health. Specifically, the severe and moderate groups exhibited higher values compared to the mild group in WC (*F* = 18.77, *p* < 0.001), and BMI (*F* = 15.78, *p* < 0.001). In addition, the number of medications (*F* = 2.96, *p* = 0.05) differed between the severe and mild groups. Compared to the mild group, both the moderate and severe groups showed poorer diabetic-specific health status in terms of HbA1c (mean HbA1c = 7.08 ± 0.76, 7.74 ± 1.33, and 7.55 ± 1.16, respectively; *F* = 2.97, *p* = 0.05), insulin (*F* = 4.09, *p* = 0.02), and insulin resistance (*F* = 4.23, *p* = 0.01, Table 3). No significant differences were observed in diabetic medications.

**Table 3:** Between-sarcopenic obesity clusters differences in demographic and clinical characteristics of older adults with type 2 diabetes.

Assessment domain	Variables	Cluster 1 (*N* = 37): Severe condition	Cluster 2 (*N* = 32): Moderate condition	Cluster 3 (*N* = 31): Mild condition	*F*-statistic (*p* value) or Chi-square (*p* value)
Demographic characteristics	Age: mean (SD)	70.54 (5.22)	69.43 (3.64)	71.54 (4.73)	1.65 (0.19)
	Sex: females, *n* (%)	22 (36.66)	21 (35.00)	17 (28.33)	0.31 (0.57)
	Sex: males, *n* (%)	15 (37.50)	11 (27.55)	14 (35.00)	0.17 (0.67)
	School years: mean (SD)	15.00 (3.68)	15.03 (3.60)	14.84 (2.80)	0.02 (0.97)
	Economic status, not that good and moderate, *n* (%)	12 (32.43)	11 (34.37)	8 (25.80)	0.30 (0.57)
	Marital status, living alone: *n* (%)	16 (43.24)	13 (40.62)	9 (29.03)	1.44 (0.22)
General health and clinical data	Health general – very good and good: *n* (%)	17 (45.94)	22 (68.75)	19 (61.29)	3.51 (0.06)
	Waist circumference (cm): mean (SD)	117.81 (14.95)^b,c^	108.35 (12.07)^a,c^	99.89 (7.08)^a,c^	**18.77 (<0.001)**
	Body mass index (kg/m^2^): mean (SD)	34.78 (6.56)^b,c^	31.91 (4.94)^a,c^	27.65 (3.34)^a,c^	**15.78 (<0.001)**
	Medications, number: mean (SD)	6.43 (2.65)^c^	5.75 (3.02)	4.97 (2.53)^a^	**2.96 (0.05)**
	Creatinine (mg/dL)	0.83 (0.20)	0.88 (0.23)	0.86 (0.19)	0.58 (0.56)
	Insulin-like growth factor 1, microgram: mean (SD)	101.21 (28.62)	103.29 (31.32)	102.87 (31.03)	0.04 (0.95)
	Vitamin D (ng/mL)	25.94 (11.16)	27.14 (10.65)	30.72 (11.26)	1.54 (0.21)
Diabetic specific measures	Diabetic age: mean (SD)	57.11 (10.46)	57.40 (8.41)	56.87 (10.08)	0.02 (0.97)
	Glucose (mg/dL): mean (SD)	153.43 (47.84)	155.22 (46.93)	131.86 (29.01)	2.67 (0.75)
	HbA1c (%): mean (SD)	7.55 (1.16)^c^	7.74 (1.33)^c^	7.08 (0.76)^a,b^	**2.97 (0.05)**
	Insulin, International Units Per Milliliter: mean (SD)	19.05 (13.03)^c^	17.63 (9.33)^c^	11.90 (7.11)^a,b^	**4.09 (0.02)**
	Insulin user, yes: *n* (%)	11 (29.72)	10 (31.25)	9 (29.03)	0.003 (0.95)
	Diabetic medications, number: mean (SD)	2.05 (1.12)	1.88 (1.12)	1.77 (0.99)	0.58 (0.56)
	GLP1 usage: *n* (%)	6 (16.21)	7 (21.87)	5 (16.12)	0.35 (0.55)
	Diabetes complications: *n* (%)	17 (45.94)	8 (25.00)	14 (45.16)	3.21 (0.07)
	Insulin resistance, HOMA-IR: mean (SD)	6.85 (45.94)^c^	6.38 (2.70)^c^	3.88 (2.91)^a,b^	**4.23 (0.01)**
Physical function	Timed Up and Go: mean (SD)	11.53 (2.04)^b,c^	10.38 (1.68)^a^	10.37 (2.15)^a^	**3.99 (0.02)**
	2 Minutes’ walk test: mean (SD)	153.01 (20.27)^b,c^	169.26 (22.12)^a^	167.57 (35.28)^a^	**4.02 (0.02)**
	Functional questionnaire, sum score: mean (SD)	13.65 (4.39)^b,c^	11.63 (2.88)^a^	11.20 (3.96)^a^	**4.01 (0.02)**
Metabolism and healthy lifestyle	RMR (kcal/day): mean (SD) - females	1633.09 (206.94)	1616.68 (233.17)	1481.41 (236.90)	2.46 (0.09)
	RMR (kcal/day): mean (SD) - males	2015.33 (289.79)	1847.00 (244.36)	1839.07 (221.86)	2.13 (0.13)
	Respiratory quotient: mean (SD)	0.78 (0.07)	0.78 (0.08)	0.77 (0.08)	0.21 (0.81)
	Energy intake (kcal/day): mean (SD)	1224.57 (585.25)	1300.80 (456.11)	1269.74 (461.16)	0.18 (0.83)
	Protein intake, g/kg body weight: mean (SD)	0.70 (0.39)	0.96 (1.29)	0.96 (1.29)	0.88 (0.41)
	Adherence to the Mediterranean diet, score: mean (SD)	9.76 (2.07)	9.41 (1.56)	9.77 (1.94)	0.39 (0.67)
	Physical activity: (Kcals/week) mean (SD)^!!^	513.15 (179.11)^c^	484.77 (175.79)^c^	901.32 (459.30)^a,b^	**4.01 (0.02)**
	Current smoke, yes: *n* (%)	1.00 (2.70)	2.00 (6.25)	2.00 (6.45)	3.22 (0.07)

^a^Statistically significantly different from “Cluster 1” (2-tailed; *p* < 0.05); ^b^Statistically significantly different from “Cluster 2” (2-tailed; *p* < 0.05); ^c^Statistically significantly different from “Cluster 3” (2-tailed; *p* < 0.05); HOMA-IR, Homeostatic Model Assessment for Insulin Resistance; SD, standard deviation; FSH, Follicle-stimulating hormone; GLP-1, Glucagon-like peptide-1; RMR, resting metabolic rate; Construction of sarcopenic obisity risk groups – risk groups classification is based on the presence of alterations in body composition (specifically appendicular mass) and muscle function (maximal knee strength test, lower extremity muscular endurance test, and mean maximal handgrip strength). A cut-off of 80% was set to determine unfavorable results, indicating that over 80% of the sample exhibited unfavorable alterations in body composition and in each muscle function test utilized (excluding handgrip test). Based on the number of unfavorable risk factors, sarcopenic obesity risk groups were categorized as follows: “severe condition” (comprising 3–4 unfavorable outcomes), “moderate condition” (with 2 unfavorable outcomes), and “mild condition” (0–1 unfavorable conditions); Calculation of sarcopenic obesity index – variables showing statistically significant differences between sarcopenic obesity risk groups were aggregated, excluding: (1) the two-minutes’ walk test and physical activity level; and (2) variables not commonly available in routine clinical practice;!! Based on a valid screener for assessing Mediterranean Diet adherence - a score ≥9 was considered as good adherence (59, 60).Values presented in bold indicate statistically significant between-group differences.

Because sex hormone levels may be influenced by age, adiposity, and glycemic control, adjusted analyses were performed using analysis of covariance; these results are presented in Table 4. After adjustment for age, BMI, and HbA1c, significant between-cluster differences in sex hormone levels were observed among females but not males. In females, FSH differed significantly across SO severity clusters (adjusted *F* = 4.95, *p* = 0.01). More specifically, post-hoc comparisons indicated that adjusted mean FSH levels were lowest in the severe cluster (38.85 ± 4.75 mIU/mL) in comparison to the mild cluster (60.18 ± 4.85 mIU/mL). Similarly, estradiol levels differed significantly between clusters in females after adjustment (adjusted *F* = 4.94, *p* = 0.01). Post-hoc analyses demonstrated that adjusted mean estradiol concentrations were higher in the severe cluster (33.41 ± 4.60 pg./mL) compared with the moderate (21.54 ± 4.20 pg./mL) and mild clusters (25.46 ± 4.10 pg./mL). No significant adjusted between-cluster differences were observed for luteinizing hormone (LH), total testosterone, or bioavailable testosterone in females. In males, none of the examined sex hormones, including FSH, estradiol, LH, total testosterone, or bioavailable testosterone, differed significantly across SO severity clusters after covariate adjustment, indicating that cluster membership was not independently associated with sex hormone levels in men.

**Table 4:** Sex hormone profiles across sarcopenic obesity severity clusters after adjustment for age, body mass index, and glycated hemoglobin.

Sex	Sex hormones	Cluster 1 (*N* = 37) - severe condition: mean (standard error)	Cluster 2 (*N* = 32) - Moderate condition: mean (standard error)	Cluster 3 (*N* = 31) - Mild condition: mean (standard error)	Adjusted *F* (*p* value)
Females	FSH, milli-international units per milliliter	38.85 (4.75)^c^	49.36 (5.28)	60.18 (4.85)^a^	**4.95 (0.01)**
	Estradiol, pictograms per milliliter	33.41 (4.60)^b,c^	21.54 (4.20)^a^	25.46 (4.10)^a^	**4.94 (0.01)**
	LH, international units per liter	19.12 (2.70)	22.39 (3.0)	24.63 (2.75)	1.02 (0.36)
	Total testosterone, nanograms per deciliter	0.17 (0.03)	0.09 (0.04)	0.10 (0.03)	1.28 (0.28)
	Bioavailable testosterone, nanograms per deciliter	0.06 (0.01)	0.01 (0.02)	0.01 (0.01)	1.89 (0.16)
Males	FSH, milli-international units per milliliter	10.80 (2.38)	6.96 (2.73)	10.95 (2.51)	0.62 (0.54)
	Estradiol, pictograms per milliliter	47.64 (5.06)	40.66 (5.58)	43.52 (5.14)	0.38 (0.68)
	LH, international units per liter	6.18 (1.47)	5.65 (1.69)	8.65 (1.55)	1.06 (0.35)
	Total testosterone, nanograms per deciliter	3.99 (0.35)	3.55 (0.41)	3.68 (0.38)	0.31 (0.73)
	Bioavailable testosterone, nanograms per deciliter	0.94 (0.07)	0.78 (0.08)	0.86 (0.08)	1.82 (0.18)

Values are adjusted means with standard errors in parentheses. Between-cluster comparisons were performed using analysis of covariance, adjusted for age, body mass index, and glycated hemoglobin. Adjusted *F* statistics and *p* values correspond to the overall cluster effect; ^a^Statistically significantly different from “Cluster 1” (2-tailed; *p* < 0.05); ^b^Statistically significantly different from “Cluster 2” (2-tailed; *p* < 0.05); ^c^Statistically significantly different from “Cluster 3” (2-tailed; *p* < 0.05); FSH, follicle-stimulating hormone; LH, luteinizing Hormone; SD, standard deviation; Construction of sarcopenic obisity risk groups – risk groups classification is based on the presence of alterations in body composition (specifically appendicular mass) and muscle function (maximal knee strength test, lower extremity muscular endurance test, and mean maximal handgrip strength). A cut-off of 80% was set to determine unfavorable results, indicating that over 80% of the sample exhibited unfavorable alterations in body composition and in each muscle function test utilized (excluding handgrip test). Based on the number of unfavorable risk factors, sarcopenic obesity risk groups were categorized as follows: “severe condition” (comprising 3–4 unfavorable outcomes), “moderate condition” (with 2 unfavorable outcomes), and “mild condition” (0–1 unfavorable conditions); Calculation of sarcopenic obesity index – variables showing statistically significant differences between sarcopenic obesity risk groups were aggregated, excluding: (1) the two-minutes’ walk test and physical activity level; and (2) variables not commonly available in routine clinical practice.Values presented in bold indicate statistically significant between-group differences.

Finally, all physical function measures were less favorable in the severe group compared to both the moderate and mild groups (range of *F*: 3.99 to 4.02, range of *p* = 0.02). Both the severe and moderate groups also demonstrated significantly lower levels of physical activity level than the mild group (*F* = 4.01, *p* = 0.02; Table 3).

### 3.3 Sarcopenic obesity risk index

In both the complete and the reduced models, the between-group SO risk index of the high and moderate risk groups compared to the low-risk group exhibited significant differences (*p* < 0.001). Finally, in the complete model, based on ROC analysis using Hanley and Mcneil methodology, employing a criterion score of >169.99, the discriminatory capability of the SO risk index approached good discriminative ability, with an AUC of 0.87 (*p* < 0.001), and sensitivity and specificity values of 76.9 and 88.5, respectively (Supplementary Figure 2a). In the reduced model, the discriminatory capability, although reduced, was moderate (AUC = 0.79, *p* < 0.001; Supplementary Figure 2b).

## 4 Discussion

The first step in SO screening involves assessing changes in skeletal function. Conforming the study’s hypothesis, this study revealed that among adults with T2D, the prevalence of altered muscle function varied depending on the type of skeletal function test. The study also aimed to identify profiles of individuals at increased risk for SO. Overall, the clusters of SO severity differed significantly in health status, physical function, and lifestyle habits. Additionally, we developed an SO index based on routine clinical measures to differentiate between various levels of SO severity. This new SO index effectively distinguished between individuals with high and mild SO risk.

### 4.1 Overall prevalence of muscle function alterations

When muscle function tests were used to assess participants, nearly all showed evidence of muscle alterations. These findings were expected, as older people have greater likelihood of having functional impairments hence at are at an increased risk of presenting with sarcopenia (30). Moreover, skeletal muscle undergoes significant structural, metabolic, and functional changes under diabetic conditions, such as muscle atrophy (31) and fiber-type transition (32), which contribute to poor muscular performance. Consequently, individuals with T2D have an estimated two to three times higher prevalence of sarcopenia compared to those without diabetes (33). Epidemiological studies report sarcopenia prevalence in T2D ranging from 7 to 29.3% across different populations (34). However, in the current study, the prevalence of sarcopenia was notably higher (90%) when any functional test was used. Based on both literature and the findings of this study, the wide variation in sarcopenia prevalence may be due to factors such as the diagnostic criteria used to assess muscle function, diabetes-related factors (e.g., microvascular and macrovascular complications), and participants’ overall lifestyle (34). These results highlight the need to standardize sarcopenic testing and enhance our understanding of the factors influencing its prevalence.

### 4.2 Differences between tests in prevalence of muscle function alterations

Prevalence of altered muscle function varied significantly across the three tests employed, with the lowest prevalence observed in HGS and the highest in STS30. These differences are not surprising, as among community-dwelling adults aged ≥ 50 years, lower extremity muscular endurance (STS30) exhibits weak correlations with upper extremity strength measured by HGS (9). Accordingly, some investigators have noted that caution should be exercised when using HGS as a proxy for lower extremity strength (35).

The lower rate of muscle function alterations in the HGS test compared to the STS30 aligns with evidence showing that lower extremity strength declines at a faster rate with age than upper extremity strength (36). The high prevalence of altered muscle function in STS30 may also be partially attributed to diabetic-related health issues, which can negatively influence test performance. Specifically, individuals with diabetes often experience complications such as diabetic peripheral neuropathy, leading to foot problems, loss of sensation, and impaired neuromotor function, all of which can reduce lower extremity muscle strength. Additionally, the STS30 tests offer several advantages over other functional tests, such as the HGS and lower extremity strength tests. The STS30 has a quick learning curve for the evaluator, uses commonly available and inexpensive materials, and can be completed in under a minute (37). Given these benefits, the STS30 may be particularly useful tool for sarcopenia screening in busy clinical settings.

### 4.3 Sarcopenic obesity severity clusters

Reported prevalence rates of SO among individuals with T2D vary due to differences in SO definitions across studies. However, a recent meta-analysis on SO prevalence in patients with T2D reported a prevalence of 27% (38), which is much lower than the 69% observed in the current study. Several factors may contribute to the higher prevalence observed in this study. Firstly, there was a higher proportion of females (60%) compared to males (40%) in this study, whereas the meta-analysis reported a female prevalence of 49%. Since SO is more prevalent in females (38), this demographic difference may partially explain the higher SO prevalence. Second, the clinical characteristics of participants in this study differed from those in the meta-analysis. For example, the mean BMI in the diabetic group was 26.4 (5.5) kg/m^2^ in the meta-analysis, compared to 31.65 (5.95) kg/m^2^ in this study. Third, this study used three different tests to define sarcopenia, classifying participants with alterations in any of the tests as having sarcopenia, which increased the prevalence of sarcopenia and SO. Additionally, this study used BIA to assess body composition, a method associated with the higher rates of SO (38).

### 4.4 Sarcopenic obesity severity clusters—between-clusters differences

SO severity clusters differed in terms of participants’ health status as well as physical function and healthy lifestyle habits (namely, physical activity level). In terms of health, anthropometric measures like BMI and WC were highest in severe cluster, followed by the moderate and by the mild clusters. These results align with previous studies showing that BMI and WC were reliable obesity indicators (39). In addition, the severe cluster had significantly more prescribed medications compared to the mild condition clusters. Similarly, a recent population-based study (*N* = 1,079) reported that SO is associated with polypharmacy (≥5 drugs) (40). These results are not surprising, as SO involves high adiposity coexisting with low muscle mass and function, leading to increased comorbidities and medication use (41). Interestingly, only glycemic control measures (HbA1c and insulin level), and not the prevalence of diabetes-related medications, differed between the SO clusters. This suggests that poorer diabetes control correlates with increased SO severity, regardless of diabetic medication usage.

In the current study no differences between-clusters in testosterone levels were observed among males across the clusters. This may be because the mean testosterone levels in all the three clusters were within the normal range. In women, estradiol, a potent estrogen hormone, plays a role in muscle health, as skeletal muscle possesses specific estradiol receptors at the fiber levels (42). Interestingly, estradiol levels in females in the severe group were higher than in the other two groups. These elevated estradiol levels may be linked to their higher obesity level (high BMI), as adipose tissue produces estrogen. In addition, FSH levels in the severe group were lower than in the other two groups. To our knowledge, few studies have explored associations between FSH and SO-related variables, such as muscle mass in females (43). Further research on the relationship between sex hormones and SO in both males and females is warranted.

Regarding physical function, sarcopenia and obesity individually reduce physical performance (44, 45), and when co-existing (SO), their combined effect is expected to further impair physical performance (46). Accordingly, in the current study, participants in the severe cluster showed poorer performance across all physical performance measures compared to the other two SO clusters. However, previous studies have produced conflicting results regarding the relationship between SO and physical function. For instance, Chang et al. (46) found that older men and women with SO had significantly lower HGS and gait speed compared to those with either sarcopenia or obesity alone. In contrast, Meng et al. (47) observed no significant difference in gait speed among men aged ≥80 years across different body composition groups, including SO. Similarly, Bouchard et al. (48) in a cohort study of 904 community-dwelling older individuals, showed that the SO group did not exhibit lower exercise capacity compared to the pure obesity group. Discrepancies between studies may be due to methodological differences in assessing obesity (BIA, BMI, Dual Energy X-ray Absorption), sarcopenia, and physical function (self-report questionnaires vs. objective methods) (49). In the current study, both objective (TUG and 2 min’ walk test) and subjective assessments were used. These findings help clarify the conflicting results in the literature by showing that lower physical function was observed only in the severe group, consisting of participants with SO and alterations in muscle function in two tests. Conversely, the group with SO showing alterations in only one muscle function test (STS30) did not differ from the non-SO group (the mild cluster). Therefore, our study indicates that the extent of muscle function alterations influences changes in physical function.

Finally, participants in mild condition had significantly higher physical activity level than those in the two more severe SO clusters. Similarly, previous studies have reported that low levels of physical activity contribute to SO (50, 51). Accordingly, for SO prevention, it is recommended for males and females engage in physical activity with energy expenditure of >3,032 kcal/week (433 kcal/day) and 2,730 kcal/week (390 kcal/day), respectively (52). More specifically, based on current guidelines and epidemiological evidence, individuals with SO should be encouraged to engage in regular aerobic physical activity for at least 150–300 min per week at moderate to vigorous intensity. Importantly, individuals performing more than 300 min per week of aerobic activity were less likely to exhibit sarcopenia compared with those meeting, but not exceeding, the World Health Organization recommendation of 150–300 min per week (53). Resistance training is also a key component in the management of SO and should be performed 2–3 days/week on nonconsecutive days. Recommended intensity ranges from 60–70% of one-repetition maximum (or rate of perceived exertion 5–6), with progression to 71–85% of one-repetition maximum (rate of perceive exertion 7–8) as tolerated. Training volume should begin with 1–2 sets of 12–15 repetitions, progressing to 3–4 sets of 6–10 repetitions, with 1–3 min of rest between sets. Programs should include 8–10 full-body exercises, using bodyweight movements, resistance bands, machines, or free weights. Progressive overload and individualized load adjustment are essential to optimize muscle strength, mass, and functional outcomes in this population (54). Finally, combined aerobic and resistance training appears to provide superior benefits compared with aerobic training alone for improving physical function in individuals with SO (55). A typical combined session lasts 40–50 min and includes approximately 5 min of warm-up, followed by 15–20 min of aerobic exercise and 15–20 min of resistance training, and concludes with 5 min of cool-down. When participants are unable to complete the full protocol, it is recommended to take 5–10 min rest after the aerobic training. This combined approach supports both cardio-metabolic health and muscle function and is recommended as an effective, time-efficient intervention strategy (56).

### 4.5 Sarcopenic obesity index discriminative ability

In this study, we found that commonly used clinical outcomes can effectively differentiate between individuals at increased SO risk (moderate and severe clusters) and those with mild risk (mild cluster). Several other studies have also successfully used various measures to distinguish between individuals with and without SO, though their reported AUCs were lower than those observed in our study. For instance, a cross-sectional study of 2,031 participants aged 70–84 years in the Korean Frailty and Aging Cohort Study found that the weight-adjusted waist index provided the best diagnostic power for SO in men, with an AUC of 0.78, while no significant associations were found in women (57). Another study examined the association between the *z*-score of the log-transformed Body Shape Index and SO in 40,468 adults (≥20 years), reporting an overall AUC of 0.735 for SO (58). Considering the AUCs reported in previous and in our current study, the newly developed SO scale from our research appears to be a valuable tool for SO screening. However, when constructing the SO index, it is important to acknowledge that medication counts and polypharmacy must be handled with caution. The validity of the proposed index depends on adherence to the predefined medication-counting approach described in the Materials and Methods section, which restricts inclusion to cardiometabolic- and diabetes-related drug families and excludes non-related therapies and supplements. This standardized approach was implemented to reduce bias arising from variability in prescribing practices and to ensure that medication burden reflects clinically meaningful disease severity and treatment complexity rather than nonspecific medication use. Accordingly, the index should be interpreted within the context of these methodological considerations.

The main limitation of the study is the relatively small sample size (*n* = 100) and the cross-sectional design, which limits the generalizability of the findings. Another limitation is that the study focused solely on the impact of various muscle function tests on SO, without examining the influence of different body composition measurement methods, which could affect SO prevalence. In addition, each muscle function test evaluated has several published cutoff points to determine alterations in function. This study did not investigate how using different cutoff points might influence the prevalence of muscle function alterations and SO clusters. Nonetheless, we used Donini et al.’s (4) recommended cutoff points, which are based on the ethnicity of the population. Finally, another important limitation of the present study is the exclusion of participants treated with SGLT-2 inhibitors. This exclusion was inherited from the design of the parent intervention study, on which the current analysis is based and from which only baseline data were used. The original trial aimed to examine the effects of a modified plant-based Mediterranean (vegeterranean) diet, circuit resistance training, and empagliflozin—alone or in combination—on metabolic and physical outcomes in older adults with T2D. To prevent pharmacological overlap and contamination with the empagliflozin intervention arm, individuals already treated with SGLT-2 inhibitors were excluded at recruitment. Although the present analysis focuses exclusively on baseline characteristics, retaining the original inclusion and exclusion criteria was necessary to ensure cohort homogeneity and methodological consistency with the parent study. Nevertheless, given the widespread contemporary use of SGLT-2 inhibitors, this exclusion may limit the generalizability of the findings, and results should be interpreted accordingly.

Despite these limitations, the study has notable strengths. We employed valid and meticulous measurement methods to assess both muscle function and body composition. Additionally, we collected a wide range of explanatory variables relevant to the development and severity of muscle function alterations and SO among older adults with T2D.

## 5 Conclusion

In this cohort of community-dwelling older adults with T2D, SO was highly prevalent, with nearly three-quarters of participants exhibiting concurrent impairments in muscle function and body composition. Alterations in appendicular lean mass and maximal knee strength emerged as the most influential markers of disease severity, while handgrip strength alone showed limited discriminatory value. These findings support a targeted clinical approach for older adults with T2D, emphasizing routine screening of lower-limb strength, functional performance (e.g., sit-to-stand), and body composition rather than reliance on single strength measures. Clinically, individuals identified as moderate-to-severe SO should be prioritized for combined aerobic and progressive resistance training, with particular focus on lower-extremity strength, alongside strategies addressing central adiposity and glycemic control. Implementing such structured, severity-informed exercise recommendations may improve physical function, metabolic health, and overall risk stratification in elderly patients with SO.

## Data Availability

The raw data supporting the conclusions of this article will be made available by the authors, without undue reservation.
