# Supplementary material for: Variability in muscle function deficits and clinical identification of sarcopenic obesity in older adults with type 2 diabetes
Source: Front Med (Lausanne). 2026 Feb 18;12:1747916. doi: 10.3389/fmed.2025.1747916 (PMC12956641; doi:10.3389/fmed.2025.1747916)
Supplement: Supplementary file 1 [file Table_1.docx]

**Supplementary figure 1**

**
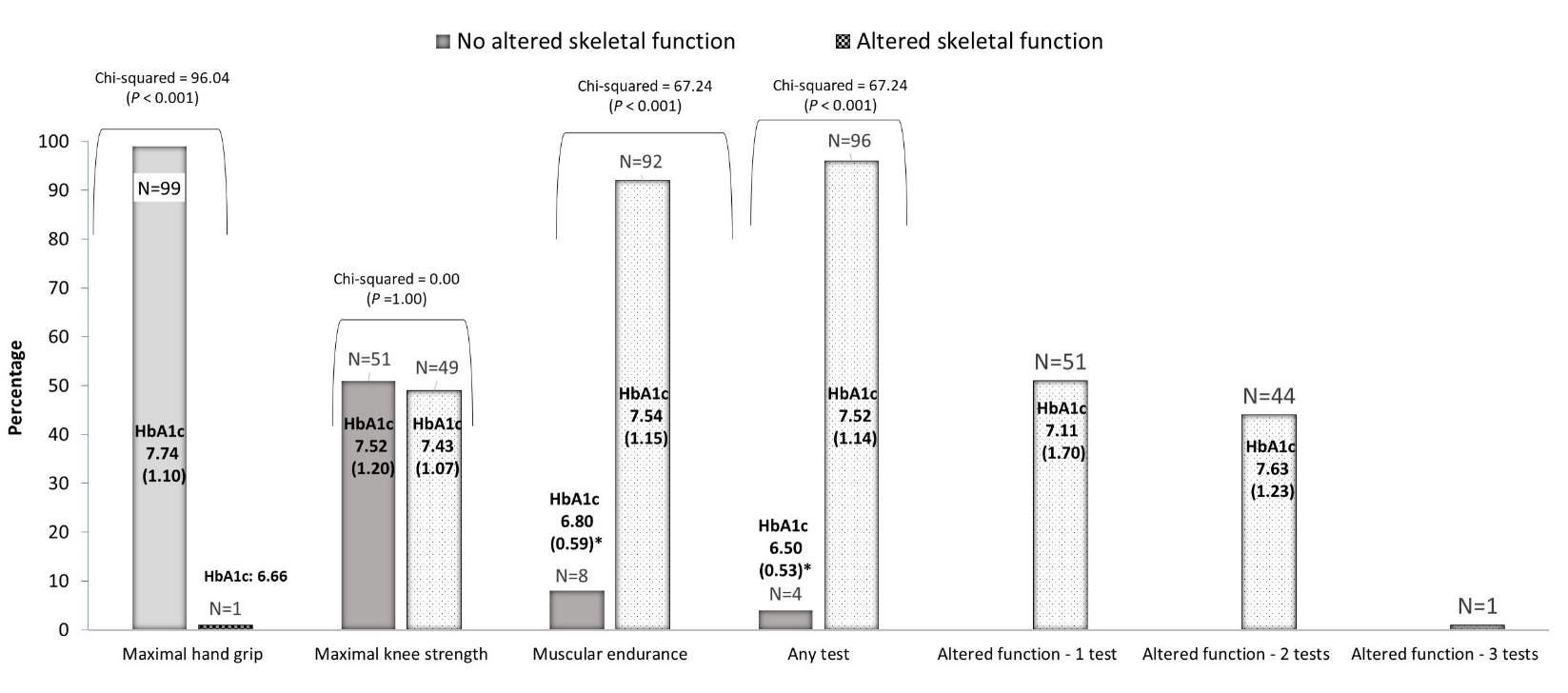
Prevelance of altered muscle function in the three muscular functional tests in relation to hemoglobin A1C levels.**

*Notes*: Muscle function impairment varied across tests: 1% in handgrip strength, 49% in knee strength, and 92% in sit-to-stand. Impairment was found in one (51%), two (44%), or all three tests (1%), while 4% showed no impairment; * significant differences between "no altered skeletal function" and "altered skeletal function" groups; HbA1c, Hemoglobin A1C; *Maximal handgrip* **-** Impaired HGS was defined as maximal strength < 27 kg and < 16 Kg for males and females, respectively;[3] *Maximal knee strength* - Impaired knee extension strength defined as maximal knee extension strength/ total weight < 0.4 and <0.31 kg/kg for males and females, respectively;[3] *Muscular endurance* **–** Impaired function was categorized based on age and sex starting from <17-15 repetitions up to <13-12 repetitions.[3]

**Supplementry figure 2**

**Receiver operating characteristic analysis of sarcopenic obesity risk index: high and moderate risk groups vs. low risk group**

**
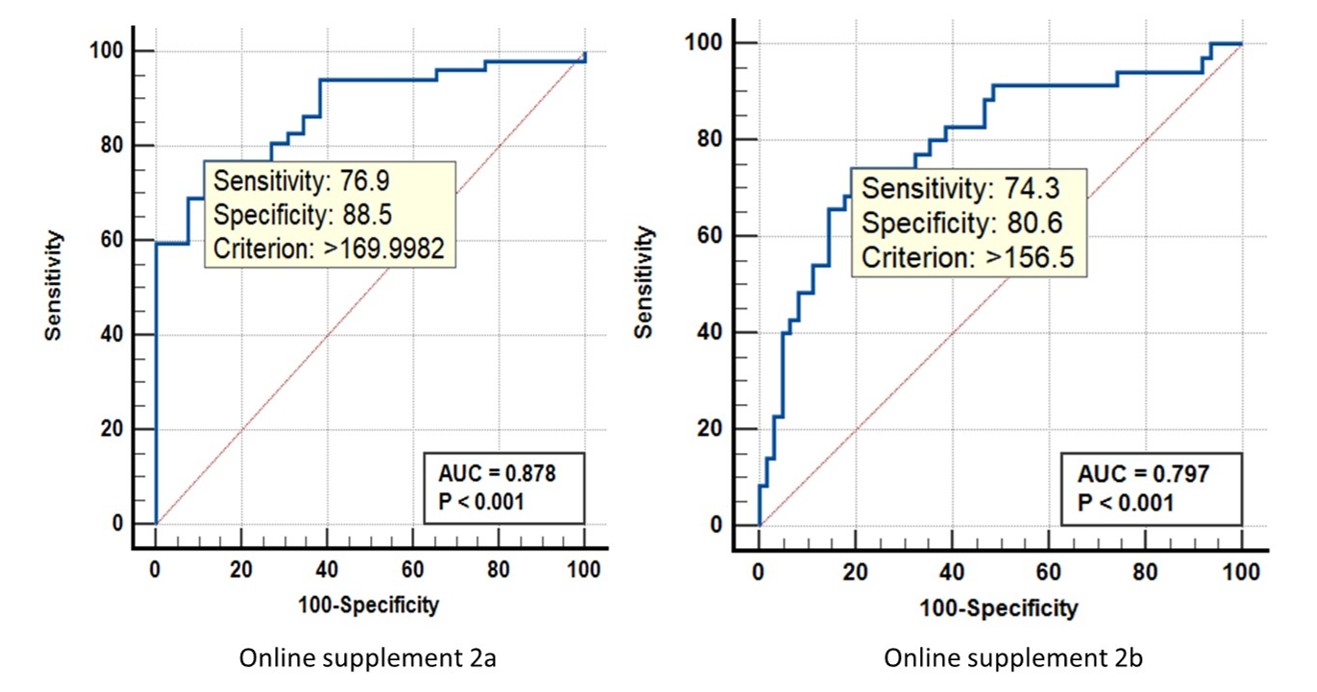
**

2a. Comlete sacropenic obesity risk index (including Timed Up and Go test)

2b. Reduced sacropenic obesity risk index (without Timed Up and Go test)

*Notes*: In the full model, the risk index showed good accuracy with 76.9% sensitivity and 88.5% specificity. The reduced model had moderate accuracy; AUC, area under the curve; In the curve, the true positive rate (sensitivity) is plotted in function of the false positive rate (100-specificity) for different cut-off points of a parameter. Each point on the curve represents a sensitivity/specificity pair corresponding to a particular decision threshold; *Construction of sarcopenic obesity risk groups* – risk groups classification is based on the presence of alterations in body composition (specifically appendicular mass) and muscle function (maximal knee strength test, lower extremity muscular endurance test, and mean maximal handgrip strength). A cut-off of 80% was set to determine unfavorable results, indicating that over 80% of the sample exhibited unfavorable alterations in body composition and in each muscle function test utilized (excluding handgrip test). Based on the number of unfavorable risk factors, sarcopenic obesity risk groups were categorized as follows: "severe condition" (comprising 3-4 unfavorable outcomes), "moderate condition" (with 2 unfavorable outcomes), and "mild condition" (0-1 unfavorable conditions).
